# Supplementary material for: Characterization of tumor heterogeneity by latent haplotypes: a sequential Monte Carlo approach
Source: PeerJ. 2018 May 30;6:e4838. doi: 10.7717/peerj.4838 (PMC5984585; doi:10.7717/peerj.4838)
Supplement: Supplemental Information 1 [file peerj-06-4838-s001.pdf]

Supplemental Material for "Characterization of tumor heterogeneity by latent haplotypes: a sequential Monte Carlo approach"

Oyetunji Ogundijo and Xiaodong Wang *

S1

Here, we present how the point estimates of the unknown variable are obtained from the posterior weighted Monte Carlo samples for the proposed SMC algorithm. We follow the procedures highlighted in [2].

First, we factorize the joint posterior distribution of all the unknown variables given the input dataset (matrices $\mathbf{Y}$ and $\mathbf{V}$) as follows:

$$p(C, \mathbf{Z}, \mathbf{W}, p|\mathbf{Y}, \mathbf{V}) = p(C|\mathbf{Y}, \mathbf{V})p(\mathbf{Z}|\mathbf{Y}, \mathbf{V}, C)p(\mathbf{W}, p|\mathbf{Y}, \mathbf{V}C, \mathbf{Z}).$$

Then, based on the available posterior Monte Carlo samples for the haplotype matrix $\mathbf{Z}$, we approximately evaluate the marginal posterior distribution for C and then determine the maximum a posteriori (MAP) estimate $\hat{C}$.

Next, conditional on $\hat{C}$, we then estimate $\mathbf{Z}$ as follows. For any two matrices $\mathbf{Z}$ and $\mathbf{Z}'$, $1 \leq c, c' \leq \hat{C}$, define

$$\mathcal{D}_{cc'}(\mathbf{Z}, \mathbf{Z}') = \sum_{t=1}^{T} |z_{tc} - z'_{tc'}|,$$

and define a distance

$$d(\mathbf{Z}, \mathbf{Z}') = \min \sum_{c=1}^{\hat{C}} \mathcal{D}_{c,\pi_c}(\mathbf{Z}, \mathbf{Z}'),$$

where π_c is a permutation of $\{1, ..., \hat{C}\}$ and the minimum is over all possible permutations. Thus, an estimate for $\mathbf{Z}$ is defined as:

$$\hat{\mathbf{Z}} = \underset{\mathbf{Z}'}{\operatorname{argmin}} \int d(\mathbf{Z}, \mathbf{Z}') dp(\mathbf{Z}|\mathbf{Y}, \hat{C})$$
$$\approx \underset{\mathbf{Z}'}{\operatorname{argmin}} \sum_{i=1}^{N} w_T^i d(\mathbf{Z}_T^i, \mathbf{Z}')$$

*to whom correspondence should be addressed

Algorithm 1 Sampling from $P(\mathbf{z}_t|\mathbf{Z}_{t-1}, \alpha)$ using the Indian Buffet Process

$\mathbf{Z} \leftarrow \mathbf{Z}_{t-1}$
if $t = 1$ **then**
  Sample $C_t^{new} \sim \text{Pois}(\alpha)$.
  Sample $\mathbf{z}_{t,1:C_t^{new}} \leftarrow 1$.
else
  $C_+ \leftarrow$ Number of non-zero columns in $\mathbf{Z}$
  for $c = 1, ..., C_+$ **do**
    $m_{-t,c} \leftarrow$ number of $1's$ in column c in Z.
    Sample $z_{t,c}$ according to $P(z_{t,c} = 1) \sim \text{Bern}\left(\frac{m_{-t,c}}{t}\right)$,
  end for
  Sample $C_t^{new} \sim \text{Pois}\left(\frac{\alpha}{t}\right)$.
  $\mathbf{z}_{t,(C_+ + 1):(C_+ + C_t^{new})} \leftarrow 1$.
end if

for posterior Monte Carlo samples, $\{\mathbf{Z}_T^i\}_{i=1}^N$ and the normalized weights $\{w_i\}_{i=1}^N$.

Finally, we report posterior estimates $\hat{\mathbf{W}}$ and $\hat{p}$ for $\mathbf{W}$ and p, respectively conditional on $\hat{C}$ and $\hat{\mathbf{Z}}$.

S2

The procedure for resampling is itemized as follows:

- Interpret each weight w_t^i as the probability of obtaining the sample index i.
- Draw N particles from the discrete probability distribution $\{w_t^i\}$ and replace the old particle set with this new one.
- Set all weights to the constant value $w_t^i = 1/N$.

S3

In addition to the results presented in the main paper for the simulated datasets, we present additional results for more combinations of T, C, S and r. The results in Table 1 shows the $e_{p_{ts}}$, e_Z and e_W computed for the proposed SMC-based, MCMC-based and MAP-based algorithms. In our experiments, we set $a_0 = 0.4$, $a = 6$, $a_{00} = 1$, and $b_{00} = 100$. α was set to 1 and 0.4 for the simulated and the CLL datasets, respectively.

S4

Here, we present the rest of the results obtained from the CLL datasets. Before presenting the results, we briefly describe the data pre-processing for the CLL

Table 1: $e_{p_{ts}}$, e_Z and e_W computed for the proposed SMC-based, MCMC-based and the MAP-based algorithms for number of SNVs $T = 20$, number of haplotypes $C = 4$, number of samples $S = 5$ and $r \in \{20, 40, 50, 200, 1000, 10000\}$.

$T = 20$, $C = 4$ and $S = 5$									
	SMC-based			MCMC-based			MAP-based		
r	$e_{p_{ts}}$	e_Z	e_W	$e_{p_{ts}}$	e_Z	e_W	$e_{p_{ts}}$	e_Z	e_W
20	0.0133	0.1025	0.0401	0.1437	0.1580	0.1000	0.1309	0.1520	0.0900
40	0.0200	0.0085	0.0300	0.1400	0.1250	0.0980	0.1305	0.1305	0.1101
50	0.0134	0.0085	0.0209	0.0835	0.0612	0.0705	0.0715	0.0505	0.0800
200	0.0102	0.0091	0.0099	0.0706	0.0395	0.0328	0.0627	0.0425	0.0217
1000	0.0103	0.0000	0.0290	0.0500	0.0055	0.0220	0.0621	0.0060	0.0435
10000	0.0012	0.0000	0.0030	0.0216	0.0025	0.0100	0.310	0.0045	0.0122

datasets. DNA and RNA sequencing was done to an average depth of 40×, sequence reads were aligned to human GRCh37.1 reference genome, somatic mutations were identified and annotated, and the VAFs were calculated (details in [3]). In addition, targeted sequencing were performed on selected somatic substitution sites in protein-coding genes from genomic DNA. The targeted sequencing was done to an average depth of $100,000\times$ (details in [3]). The complete datasets for the WGS and the deeply sequenced somatic mutations are in [3] and the deeply sequenced datasets used in all the analyses in this paper are in Tables 23 - 28.

In the main paper, we presented part of the results obtained from analyzing the CLL datasets with the proposed SMC algorithm. Here, we present the rest of the results for the CLL003, CLL0077 and CLL006, and the results obtained when the CLL datasets are analyzed with the MCMC-based and the MAP-based algorithms. The posterior point estimates of the matrix of haplotypes **Z** and the matrix of proportions **W** are presented in Tables 2 - 16.

S5

In this section, we provide the results obtained on each of the CLL datasets with manual analysis carried out by [3] and when analyzed with the method in [1] (Phylosub). The results are shown in Tables 17 - 22. In the genotype matrices, each column denotes a subclone, as opposed to the haplotype in our analyses, and a 0 and a 1 denote the presence and absence of a mutation in a subclone, respectively. Also, the clonal proportion matrices show the proportions of each subclone in each of the samples.

Table 2: *CLL003*: Estimate of the proportions of the haplotypes in each sample using the proposed SMC-based algorithm.

	Samples				
Haplotype	**a**	**b**	**c**	**d**	**e**
C0	0.0031	0.0036	0.0302	0.0174	0.0087
C1	0.0226	0.0225	0.0569	0.2437	0.3347
C2	0.3978	0.4189	0.0822	0.0265	0.0287
C3	0.0610	0.0596	0.1787	0.1821	0.1196
C4	0.0244	0.0262	0.1947	0.2246	0.1536
C5	0.1116	0.0540	0.0994	0.0547	0.0240
C6	0.3794	0.4150	0.3580	0.2510	0.3307

a indicates before chlorambucil; **b** indicates before fludarabine, cyclophosphamide, rituximab; **c** indicates immediately after 6 cycles of fludarabine, cyclophosphamide, rituximab; **d** indicates before ofatumumab; **e** indicates after ofatumumab [3].

References

[1] Jiao,W., et al. (2014) Inferring clonal evolution of tumors from single nucleotide somatic mutations. *BMC bioinformatics*, **15**, 35.

[2] Lee,J., et al. (2015) A Bayesian feature allocation model for tumor heterogeneity. *The Annals of Applied Statistics*, **9**, 621–639.

[3] Schuh,A., et al. (2012) Monitoring chronic lymphocytic leukemia progression by whole genome sequencing reveals heterogeneous clonal evolution patterns. *Blood*, **120**, 4191–4196.

Table 3: *CLL077*: Estimate of the proportions of the haplotypes in each sample using the proposed SMC-based algorithm.

	Samples				
Haplotype	**a**	**b**	**c**	**d**	**e**
C0	0.0046	0.0100	0.0315	0.0156	0.0171
C1	0.0450	0.0597	0.0518	0.1428	0.0314
C2	0.1272	0.0885	0.0793	0.1159	0.0905
C3	0.0144	0.0196	0.0360	0.0465	0.1333
C4	0.1222	0.0750	0.0814	0.0992	0.0274
C5	0.1820	0.2600	0.2702	0.1819	0.0581
C6	0.3099	0.2917	0.2423	0.2691	0.4752
C7	0.0779	0.1216	0.0471	0.0537	0.0622
C8	0.0789	0.0308	0.0995	0.0412	0.0344
C9	0.0378	0.0431	0.0611	0.0341	0.0703

a indicates before chlorambucil; **b** indicates before fludarabine, cyclophosphamide; **c** indicates immediately after 4 cycles of fludarabine, cyclophosphamide; **d** indicates before ofatumumab; **e** indicates relapse 9 months after ofatumumab [3].

Table 4: *CLL006*: Estimate of the proportions of the haplotypes in each sample using the proposed SMC-based algorithm.

	Samples				
Haplotype	**a**	**b**	**c**	**d**	**e**
C0	0.0168	0.0207	0.0181	0.0086	0.0183
C1	0.0438	0.1112	0.0676	0.1138	0.1143
C2	0.0887	0.1138	0.1446	0.0554	0.0600
C3	0.1016	0.0804	0.1007	0.2093	0.1947
C4	0.0701	0.0743	0.0910	0.0381	0.0643
C5	0.1321	0.1118	0.0682	0.0698	0.0514
C6	0.1567	0.1036	0.0591	0.0956	0.0855
C7	0.1598	0.1016	0.1216	0.0821	0.0873
C8	0.0942	0.1031	0.1804	0.1098	0.1485
C9	0.0503	0.0789	0.1003	0.1227	0.0419
C10	0.0859	0.1007	0.0484	0.0947	0.1337

a indicates before fludarabine, cyclophosphamide; **b** indicates before Rituximab, cyclophosphamide, rituximab; **c** indicates before Ofatmumab; **d** indicates immediately after Ofatumumab; **e** indicates relapse 12 months after Ofatumumab [3].

Table 5: *CLL003*: Estimates of the mutational profiles of haplotypes **Z** in the samples using the MCMC-based algorithm.

Gene	C1	C2	C3	C4	C5	C6
ADAD1	1	1	1	1	1	1
AMTN	0	1	0	0	0	0
APBB2	1	1	0	0	0	0
ASXL1	1	0	0	1	0	0
ATM	0	1	0	0	1	0
BPIL2	0	1	0	0	0	0
CHRNB2	1	0	0	1	0	0
CHTF8	1	1	1	0	0	0
FAT3	1	0	1	1	0	0
HERC2	1	1	1	0	0	1
IL11RA	1	1	1	0	0	0
MTUS1	0	1	0	0	0	0
MUSK	1	0	0	1	0	0
NPY	1	0	0	1	0	0
NRG3	1	0	0	1	0	0
PLEKHG5	0	1	0	0	0	0
SEMA3E	1	0	0	1	0	0
SF3B1	1	0	1	0	0	0
SHROOM1	1	1	1	0	0	0
SPTAN1	1	1	0	0	0	0

The genes where the mutations are found are shown in the first column.

Table 6: *CLL003*: Posterior point estimate of the proportions of the haplotypes in each sample using the MCMC-based algorithm.

	Samples				
Haplotype	**a**	**b**	**c**	**d**	**e**
C0	0.0041	0.0014	0.0320	0.0152	0.0020
C1	0.0645	0.0259	0.0445	0.2545	0.3607
C2	0.2995	0.4501	0.0537	0.0332	0.0445
C3	0.0813	0.0711	0.1450	0.1998	0.1223
C4	0.0400	0.0345	0.2001	0.2555	0.1845
C5	0.1453	0.0445	0.0899	0.0334	0.0441
C6	0.3653	0.3725	0.4358	0.2084	0.2419

Table 7: *CLL077*: Estimates of the mutational profiles of haplotypes **Z** in the samples using the MCMC-based algorithm.

Gene	C1	C2	C3	C4	C5	C6	C7	C8
BCL2L13	1	0	1	1	1	1	1	0
COL24A1	0	0	1	0	0	0	0	0
DAZAP1	0	0	0	1	1	0	0	0
EXOC6B	0	0	0	1	1	0	0	1
GHDC	1	0	0	1	1	0	0	1
GPR158	1	0	1	1	1	0	0	1
HMCN1	0	0	0	0	0	0	0	0
KLHDC2	0	0	1	0	0	0	0	0
LRRC16A	0	0	0	0	1	0	0	0
MAP2K1	0	0	1	0	0	0	0	0
NAMPT	1	0	1	1	1	0	1	0
NOD1	0	0	1	0	0	0	0	0
OCA2	0	0	0	1	1	0	0	1
PLA2G16	0	0	0	1	1	0	1	0
SAMHD1	0	1	1	1	1	0	1	0
SLC12A1	0	1	1	1	1	0	0	0

Table 8: *CLL077*: Posterior point estimate of the proportions of the haplotypes in each sample using the MCMC-based algorithm.

	Samples				
Haplotype	**a**	**b**	**c**	**d**	**e**
C0	0.0029	0.0201	0.0333	0.0117	0.0149
C1	0.0468	0.0620	0.0571	0.1632	0.0319
C2	0.1199	0.0421	0.0933	0.1232	0.0858
C3	0.0202	0.0184	0.0280	0.0505	0.1555
C4	0.1334	0.0512	0.0795	0.0901	0.0234
C5	0.2775	0.2710	0.3001	0.2011	0.0473
C6	0.2782	0.3541	0.2999	0.2723	0.5056
C7	0.0811	0.1311	0.0499	0.0499	0.0800
C8	0.0421	0.0500	0.0589	0.0380	0.0711

Table 9: *CLL006*: Estimates of the mutational profiles of haplotypes **Z** in the samples using the MCMC-based algorithm.

Gene	C1	C2	C3	C4	C5	C6	C7	C8	C9
ARHGAP29	1	1	1	1	0	1	0	1	0
EGFR	1	1	1	1	1	0	0	0	0
IRF4	1	0	1	0	0	0	0	0	0
KIAA0182	1	1	1	0	1	0	1	0	0
KIAA0319L	1	1	1	1	0	0	0	1	0
KLHL4	1	1	0	0	1	1	1	1	1
MED12	1	1	1	1	1	1	1	0	1
PILRB	1	1	1	0	1	0	1	0	0
RBPJ	1	0	0	0	0	0	0	0	0
SIK1	1	1	1	1	1	0	0	0	0
U2AF1	1	1	1	0	1	1	0	0	1

The genes where the mutations are found are shown in the first column.

Table 10: *CLL006*: Posterior point estimate of the proportions of the haplotypes in each sample using MCMC.

	Samples				
Haplotype	**a**	**b**	**c**	**d**	**e**
C0	0.0115	0.0199	0.0099	0.0023	0.0115
C1	0.0523	0.1204	0.0676	0.1087	0.1093
C2	0.0782	0.1207	0.1740	0.0801	0.0411
C3	0.1532	0.0911	0.1115	0.3000	0.2234
C4	0.0988	0.0785	0.0699	0.0344	0.0522
C5	0.1524	0.1800	0.0884	0.0588	0.0622
C6	0.1677	0.1005	0.0891	0.0834	0.0935
C7	0.1145	0.1106	0.1149	0.0733	0.0588
C8	0.1211	0.1000	0.1732	0.1022	0.1600
C9	0.0503	0.0783	0.1015	0.1568	0.1880

Table 11: *CLL003*: Estimates of the mutational profiles of haplotypes $\mathbf{Z}$ in the samples using the MAP-based algorithm.

Gene	C1	C2	C3	C4	C5
ADAD1	1	1	1	1	1
AMTN	0	1	0	0	0
APBB2	1	1	0	0	0
ASXL1	1	0	0	1	0
ATM	0	1	0	0	1
BPIL2	0	1	0	0	0
CHRNB2	1	0	0	1	0
CHTF8	1	1	1	0	0
FAT3	1	0	1	1	0
HERC2	1	1	1	1	0
IL11RA	1	1	1	0	0
MTUS1	0	1	0	0	0
MUSK	1	0	0	1	0
NPY	1	0	0	1	0
NRG3	1	0	0	1	0
PLEKHG5	0	1	0	0	0
SEMA3E	1	0	0	1	0
SF3B1	1	0	1	0	0
SHROOM1	1	1	1	0	0
SPTAN1	1	1	0	0	0

The genes where the mutations are found are shown in the first column.

Table 12: *CLL003*: Posterior point estimate of the proportions of the haplotypes in each sample using the MAP-based algorithm.

	Samples				
Haplotype	**a**	**b**	**c**	**d**	**e**
C0	0.0092	0.0058	0.0910	0.0220	0.0052
C1	0.0898	0.0811	0.0825	0.3011	0.3937
C2	0.4624	0.5771	0.0537	0.0419	0.0810
C3	0.0904	0.0628	0.3655	0.2200	0.1635
C4	0.0492	0.1898	0.3221	0.3680	0.2744
C5	0.2990	0.0834	0.0852	0.0470	0.0822

Table 13: *CLL077*: Estimates of the mutational profiles of haplotypes **Z** in the samples using the MAP-based algorithm.

Gene	C1	C2	C3	C4	C5	C6	
BCL2L13	1	0	1	1	1	1	1
COL24A1	0	0	1	0	0	0	0
DAZAP1	0	0	0	1	1	0	0
EXOC6B	0	0	1	1	1	1	0
GHDC	1	0	0	1	1	0	1
GPR158	1	0	1	1	1	0	0
HMCN1	0	0	0	0	0	0	0
KLHDC2	0	0	1	0	0	0	0
LRRC16A	0	0	0	0	1	0	0
MAP2K1	0	0	1	0	0	0	0
NAMPT	1	0	1	1	1	0	1
NOD1	0	0	1	0	0	0	0
OCA2	0	0	0	1	1	1	0
PLA2G16	0	0	0	1	1	0	1
SAMHD1	0	1	1	1	1	0	1
SLC12A1	0	1	1	1	1	0	0

Table 14: *CLL077*: Posterior point estimate of the proportions of the haplotypes in each sample using the MAP-based algorithm.

	Samples				
Haplotype	**a**	**b**	**c**	**d**	**e**
C0	0.0032	0.0201	0.0108	0.0108	0.0251
C1	0.0422	0.0569	0.0422	0.2000	0.0483
C2	0.1501	0.0801	0.1083	0.1101	0.0688
C3	0.0310	0.0132	0.0501	0.0400	0.1990
C4	0.1355	0.0488	0.0499	0.0708	0.0402
C5	0.2599	0.2397	0.3500	0.2097	0.0740
C6	0.2689	0.3601	0.3301	0.3324	0.4690
C7	0.1092	0.0623	0.0586	0.0262	0.0756

Table 15: *CLL006*: Estimates of the mutational profiles of haplotypes **Z** in the samples using the MAP-based algorithm.

Gene	C1	C2	C3	C4	C5	C6	C7	C8	C9
ARHGAP29	1	1	1	1	0	1	0	1	1
EGFR	1	1	1	1	1	0	0	0	0
IRF4	1	0	1	0	0	0	0	0	0
KIAA0182	1	1	1	0	1	0	1	0	0
KIAA0319L	1	1	1	1	0	0	0	1	1
KLHL4	1	1	0	0	1	1	1	1	1
MED12	1	1	1	1	1	1	1	0	1
PILRB	1	1	1	0	1	0	1	0	0
RBPJ	1	0	0	0	0	0	0	0	0
SIK1	1	1	1	1	1	0	0	0	0
U2AF1	1	1	1	0	1	1	0	0	1

The genes where the mutations are found are shown in the first column.

Table 16: *CLL006*: Posterior point estimate of the proportions of the haplotypes in each sample using the MAP-based algorithm.

	Samples				
Haplotype	**a**	**b**	**c**	**d**	**e**
C0	0.0122	0.0144	0.0101	0.0080	0.0102
C1	0.0479	0.1411	0.0800	0.1000	0.1000
C2	0.0821	0.1000	0.1489	0.0400	0.0334
C3	0.1343	0.0602	0.1199	0.2644	0.2988
C4	0.0800	0.0822	0.0409	0.0582	0.0401
C5	0.1629	0.2001	0.0884	0.0633	0.0400
C6	0.1420	0.0802	0.0900	0.0599	0.0602
C7	0.0907	0.1001	0.1301	0.0801	0.0445
C8	0.1596	0.0808	0.1600	0.1010	0.1722
C9	0.0883	0.1409	0.1317	0.2251	0.2006

Table 17: CLL003: Clonal genotypes.

		Manual				Phylosub			
	Gene	C1	C2	C3	C4	C1	C2	C3	C4
1	ADAD1	1	1	1	1	1	1	1	1
2	AMTN	0	1	0	0	0	1	0	0
3	APBB2	0	1	0	0	0	1	0	0
4	ASXL1	1	0	0	1	1	0	0	1
5	ATM	0	1	0	0	0	1	0	0
6	BPIL2	0	1	0	0	0	1	0	0
7	CHRNB2	1	0	0	0	1	0	0	0
8	CHTF8	1	1	1	1	1	1	1	1
9	FAT3	1	0	0	0	1	0	0	0
10	HERC2	1	1	1	1	1	1	1	1
11	IL11RA	1	1	1	1	1	1	1	1
12	MTUS1	0	1	0	0	0	1	0	0
13	MUSK	1	0	0	1	1	0	0	1
14	NPY	1	0	0	0	1	0	0	0
15	NRG3	1	0	0	0	1	0	0	0
16	PLEKHG5	0	1	0	0	0	1	0	0
17	SEMA3E	1	0	0	1	1	0	0	1
18	SF3B1	1	1	1	1	1	1	1	1
19	SHROOM1	1	1	1	1	1	1	1	1
20	SPTAN1	0	1	0	0	0	1	0	0

Table 18: CLL003: Clonal proportions.

		a	b	c	d	e
Manual	C0	0.06	0.02	0.30	0.06	0.02
	C1	0.00	0.01	0.44	0.89	0.96
	C2	0.82	0.91	0.13	0.00	0.00
	C3	0.03	0.00	0.00	0.00	0.00
	C4	0.09	0.06	0.13	0.05	0.03
Phylosub	C0	0.08	0.00	0.36	0.08	0.01
	C1	0.00	0.00	0.43	0.92	0.99
	C2	0.79	0.86	0.11	0.00	0.00
	C3	0.00	0.07	0.00	0.00	0.01
	C4	0.13	0.07	0.10	0.00	-0.01

Table 19: CLL077: Clonal genotypes.

		Manual				Phylosub			
	Gene	C1	C2	C3	C4	C1	C2	C3	C4
1	BCL2L13	1	1	1	1	1	1	1	1
2	COL24A1	0	1	0	0	0	1	0	0
3	DAZAP1	0	0	1	1	0	0	1	1
4	EXOC6B	0	0	1	1	0	0	1	1
5	GHDC	0	0	1	1	0	0	1	1
6	GPR158	1	1	1	1	1	1	1	1
7	HMCN1	0	1	0	0	0	1	0	0
8	KLHDC2	0	1	0	0	0	1	0	0
9	LRRC16A	0	0	0	1	0	0	0	1
10	MAP2K1	0	1	0	0	0	1	0	0
11	NAMPTL	1	1	1	1	1	1	1	1
12	NOD1	0	1	0	0	0	1	0	0
13	OCA2	0	0	1	1	0	0	1	1
14	PLA2G16	0	0	1	1	0	0	1	1
15	SAMHD1	1	1	1	1	1	1	1	1
16	SLC12A1	1	1	1	1	1	1	1	1

Table 20: CLL077: Clonal proportions.

		**a**	**b**	**c**	**d**	**e**
Manual	C0	0.08	0.03	0.00	0.04	0.38
	C1	0.20	0.15	0.16	0.17	0.08
	C2	0.00	0.03	0.04	0.14	0.31
	C3	0.39	0.27	0.30	0.26	0.13
	C4	0.33	0.52	0.50	0.39	0.10
Phylosub	C0	0.09	0.03	0.01	0.04	0.40
	C1	0.15	0.17	0.17	0.17	0.04
	C2	0.00	0.03	0.03	0.13	0.32
	C3	0.39	0.19	0.33	0.28	0.16
	C4	0.37	0.58	0.46	0.38	0.08

Table 21: CLL006: Clonal genotypes.

		Manual					Phylosub					
	Gene	C1	C2	C3	C4	C5	C1	C2	C3	C4	C5	C6
1	ARHGAP29	1	1	1	1	1	1	1	1	1	1	1
2	EGFR	1	1	1	1	1	1	1	1	1	1	0
3	IRF4	1	0	0	0	1	1	0	0	0	1	0
4	KIAA0182	1	1	1	1	1	1	1	1	1	1	1
5	KIAA0319L	1	0	0	1	1	1	0	0	1	1	0
6	KLHL4	1	1	1	1	1	1	1	1	1	1	1
7	MED12	1	1	1	1	1	1	1	1	1	1	1
8	PILRB	1	1	1	1	1	1	1	1	1	1	1
9	RBPJ	1	0	0	0	0	1	0	0	0	0	0
10	SIK1	1	1	1	1	1	1	1	1	1	1	1
11	U2AF1	1	1	0	1	1	1	1	0	1	1	0

Table 22: CLL006: Clonal proportions.

		a	b	c	d	e
Manual	C0	0.00	0.00	0.00	0.00	0.00
	C1	0.02	0.09	0.03	0.13	0.13
	C2	0.31	0.31	0.32	0.22	0.17
	C3	0.32	0.09	0.30	0.06	0.09
	C4	0.11	0.08	0.11	0.15	0.04
	C5	0.24	0.43	0.24	0.44	0.57
Phylosub	C0	0.00	0.00	0.00	0.00	0.00
	C1	0.02	0.08	0.03	0.14	0.13
	C2	0.33	0.37	0.36	0.22	0.16
	C3	0.23	0.05	0.20	0.04	0.05
	C4	0.13	0.03	0.08	0.17	0.05
	C5	0.23	0.45	0.26	0.41	0.56
	C6	0.06	0.02	0.07	0.02	0.05

Table 23: CLL003: Variant Count (matrix $\mathbf{Y}$) obtained via targeted deep sequencing ($r = 100,000\times$).

	Gene	Chromosome	**a**	**b**	**c**	**d**	**e**	Normal control
1	ADAD1	chr4	52442	46444	44095	50480	50214	00216
2	AMTN	chr4	06622	06933	00975	00045	00037	00011
3	APBB2	c4.fa	29394	33730	04112	00131	00125	00025
4	ASXL1	chr20	04041	02544	23617	37246	41221	00095
5	ATM	chr11	57014	68604	05183	00242	00321	00051
6	BPIL2	chr22	39019	31157	05224	00150	00223	00085
7	CHRNB2	chr1	00081	00328	13268	32461	36523	00090
8	CHTF8	c16.fa	51520	61061	50527	61186	64850	00260
9	FAT3	chr11	00039	00372	16610	32888	36820	00054
10	HERC2	chr15	20014	17993	14088	18078	19353	00132
11	IL11RA	chr9	50124	53084	36376	46238	47778	00215
12	MTUS1	chr8	39685	45533	06878	00498	00281	00081
13	MUSK	chr9	01006	00592	04578	07798	08259	00118
14	NPY	chr7	00042	00209	10337	22337	21358	00006
15	NRG3	c10.fa	00058	00304	13618	24075	25070	00040
16	PLEKHG5	chr1	16295	15207	02203	00075	00146	00040
17	SEMA3E	chr7	01320	00858	08095	12293	12957	00047
18	SF3B1	chr2	41887	44071	27117	47852	43946	00096
19	SHROOM1	chr5	71722	82740	46660	73472	67394	00169
20	SPTAN1	chr9	21414	25874	03605	00132	00108	00035

Table 24: CLL003: Total Count (matrix $\mathbf{V}$) obtained via targeted deep sequencing ($r = 100,000\times$).

	Gene	Chromosome	**a**	**b**	**c**	**d**	**e**	Normal control
1	ADAD1	chr4	104855	092965	134614	105728	101005	105245
2	AMTN	chr4	015240	014825	016116	014546	015260	014304
3	APBB2	c4.fa	070667	071391	063304	064333	065323	057835
4	ASXL1	chr20	072582	059240	079169	075616	079457	083298
5	ATM	chr11	076432	081557	051331	090588	100571	084683
6	BPIL2	chr22	099380	073042	087524	079246	089934	078166
7	CHRNB2	chr1	066796	048298	059132	069515	070694	076985
8	CHTF8	c16.fa	112740	124177	159245	138636	137115	125977
9	FAT3	chr11	086202	083674	069483	075128	076241	078269
10	HERC2	chr15	042650	036331	042043	041111	039544	042078
11	IL11RA	chr9	116954	114354	116960	104681	104983	127574
12	MTUS1	chr8	099431	097255	083024	098294	098702	087848
13	MUSK	chr9	021893	014109	015723	016672	016659	024664
14	NPY	chr7	045586	047758	052265	052391	047287	054890
15	NRG3	c10.fa	057537	054175	059557	053420	053376	057648
16	PLEKHG5	chr1	038613	034683	040156	035741	041009	038293
17	SEMA3E	chr7	027620	027644	030104	027191	026912	030020
18	SF3B1	chr2	086888	087944	083197	104069	090186	090011
19	SHROOM1	chr5	147752	165653	142899	161283	138259	136552
20	SPTAN1	chr9	053440	056385	062225	052645	055939	058272

Table 25: CLL077: Variant Count (matrix $\mathbf{Y}$) obtained via targeted deep sequencing ($r = 100,000\times$).

	Gene	Chromosome	**a**	**b**	**c**	**d**	**e**
1	BCL2L13	chr22	088237	110443	095955	099165	078504
2	COL24A1	chr1	000371	002251	003476	011087	024874
3	DAZAP1	chr19	040996	026647	035225	030848	008972
4	EXOC6B	chr2	077824	085138	077934	068329	024002
5	GHDC	chr17	047334	045895	066701	047403	016734
6	GPR158	chr10	034183	037812	031788	034419	022502
7	HMCN1	chr1	000471	001586	002341	006647	014522
8	KLHDC2	chr14	000891	002509	003139	011277	016352
9	LRRC16A	chr6	033114	059394	061346	042417	010346
10	MAP2K1	chr15	000446	001405	002331	007357	021929
11	NAMPTL	chr10	072896	086651	058490	063014	029711
12	NOD1	chr7	000202	001046	001649	005624	012481
13	OCA2	chr15	053930	057202	056659	054738	016557
14	PLA2G16	chr11	065807	077211	068209	069281	021064
15	SAMHD1	chr20	221550	227255	188610	239504	132810
16	SLC12A1	chr15	085098	082118	072361	082828	045217

Table 26: CLL077: Total Count (matrix $\mathbf{V}$) obtained via targeted deep sequencing ($r = 100,000\times$).

	Gene	Chromosome	**a**	**b**	**c**	**d**	**e**
1	BCL2L13	chr22	191464	219696	188700	206691	240454
2	COL24A1	chr1	145046	169058	142625	150564	141776
3	DAZAP1	chr19	107920	069581	088154	096146	079083
4	EXOC6B	chr2	218934	216229	200095	214111	215687
5	GHDC	chr17	134702	119229	164004	144915	130819
6	GPR158	chr10	078736	079011	063218	071738	073837
7	HMCN1	chr1	131927	118119	114853	104173	093965
8	KLHDC2	chr14	178172	168051	154861	165833	108988
9	LRRC16A	chr6	199374	228339	245709	218510	217182
10	MAP2K1	chr15	116242	085754	103076	099304	138271
11	NAMPTL	chr10	150223	173619	112183	127093	090566
12	NOD1	chr7	089500	086473	093976	092557	091325
13	OCA2	chr15	151242	143928	146832	168633	158326
14	PLA2G16	chr11	180856	187319	166881	206617	176475
15	SAMHD1	chr20	246648	240390	191044	254574	215490
16	SLC12A1	chr15	185324	169793	152710	178305	166783

Table 27: CLL006: Variant Count (matrix $\mathbf{Y}$) obtained via targeted deep sequencing ($r = 100,000\times$).

	Gene	Chromosome	**a**	**b**	**c**	**d**	**e**
1	ARHGAP29	chr1	060732	049586	059184	035207	056640
2	EGFR	chr7	063499	046552	056107	049740	074426
3	IRF4	chr6	013659	021773	011159	024792	029646
4	KIAA0182	chr16	040690	044182	019321	038794	040396
5	KIAA0319L	chr1	021432	031882	015742	023824	034787
6	KLHL4	chrX	103122	076787	080201	088517	092154
7	MED12	chrX	118007	109099	082486	131905	180700
8	PILRB	chr7	037719	030095	029638	040887	028352
9	RBPJ	chr4	000891	004923	002011	008251	007954
10	SIK1	chr21	023386	033607	058849	033939	038859
11	U2AF1	chr21	028334	033474	032440	056434	040853

Table 28: CLL006: Total Count (matrix $\mathbf{V}$) obtained via targeted deep sequencing ($r = 100,000\times$).

	Gene	Chromosome	**a**	**b**	**c**	**d**	**e**
1	ARHGAP29	chr1	126755	100469	121941	071263	115417
2	EGFR	chr7	135031	097485	120826	101788	157737
3	IRF4	chr6	106716	083179	083799	086440	084607
4	KIAA0182	chr16	072221	084463	037410	073050	076869
5	KIAA0319L	chr1	116386	106518	083271	066152	094465
6	KLHL4	chrX	112775	079087	084707	092076	095849
7	MED12	chrX	127079	112052	086626	137055	185970
8	PILRB	chr7	074007	058150	056286	076579	054844
9	RBPJ	chr4	076826	111139	155389	123305	119960
10	SIK1	chr21	051929	064677	116465	068393	079788
11	U2AF1	chr21	082781	073370	092894	120384	089511
